# Supplementary material for: A qualitative examination of injury prevention strategy and education in Ladies Gaelic football: Understanding the preferences of players and coaches
Source: PLoS One. 2023 Feb 15;18(2):e0281825. doi: 10.1371/journal.pone.0281825 (PMC9931138; doi:10.1371/journal.pone.0281825)
Supplement: S1 File — (DOCX) [file pone.0281825.s001.docx]

**S1 File. Relevant Interview guide questions for players and coaches**

*The purpose of this interview is to help us understand what adult members of the Ladies Gaelic football community think about injury prevention. So, to do that I’m going to ask you a few questions, however, if you don’t want to answer a question or you want to stop at any time that’s not a problem, and it won’t impact your relationship with the LGFA, DCU or anyone else.*

*Before we start do you have any questions for me?*

***Question List for Players and Coaches***

Section 1 - Views on Injury Prevention Strategy and Methods

1. What is your understanding of injury prevention in sport?
2. What do you think are the different components required in an injury prevention strategy for adult Ladies Gaelic footballers?
   1. What content should be included in a programme?
3. If an injury prevention strategy is to be successful, what is needed most?
4. How would you want injury prevention programmes to be delivered?

**Prompts (for acquiring greater general detail)*

- *When you say ____, what do you mean by that?*
- *Would you mind explaining your thoughts on implementing ____ more for me?*
- *Just going back to when you said____, can you explain that a bit more?*
- *When you say the strategy should include ____, are you talking about ____?*
- *You brought up ____, how would you like to see this put into practice?*
- *You mentioned _____, what is the most important in your opinion?*

Section 2 - Views on Increasing Levels of Injury Prevention Education

1. *You’ve brought up several different barriers and facilitators, we’re considering that education might be an important factor to address some of those points you have mentioned*. What do you think about the role of education in the injury prevention strategy of adult ladies Gaelic football?
2. What is the best way for us to educate players/coaches do you think?
3. What content needs to be the focus of injury prevention education?
4. What format should the injury prevention education take?

**Prompts (for acquiring greater general detail)*

- *You think ______, could you tell me more about this?*
- *You said that ____ should be part of the education, would you mind explaining this more?*
- *You mentioned ____ is important, could you tell me why that is?*

**Prompts will be dependent upon the ideas put forward by the participant in relation to injury prevention strategy*.

*****Supporting Content Questions for Players and Coaches***

Questions related to strategy

1. *When should it be done?*
2. *How much time should it take?*
3. *Should it be completed as a team or individually?*
4. *Who should lead the programme (if done as a team)?*
5. *Would you like to see equipment be used in the programme?*
6. *How often should this be done?*
7. *Should it be different at practice vs. at games?*
8. *Does the programme need to include anything else outside of injury prevention?*
9. *What are other demands that might get in the way of a programme’s adoption?*
10. *What resources, equipment or facilities should play a role in the programme?*
11. *Is it important that the programme is fun?*
12. *Do you think the priorities of coaches and players could affect adoption rates?*
13. *Who needs to support the programme for it to be successful in your opinion?*
14. *How could the exercises in the programme impact its uptake?*
15. *Do you think if people are more familiar with the exercises included it would be more successful?*
16. *How can coaches or other players impact the adoption of the programme?*

Questions related to injury prevention education

1. *How do you think education could be organised to reach a wider audience?*
2. *Who would you like to see teaching the injury prevention education?*

**Supporting questions will be used to introduce key topics in the interview that have not already been discussed.
