# Supplementary material for: A qualitative examination of injury prevention strategy and education in Ladies Gaelic football: Understanding the preferences of players and coaches
Source: PLoS One. 2023 Feb 15;18(2):e0281825. doi: 10.1371/journal.pone.0281825 (PMC9931138; doi:10.1371/journal.pone.0281825)
Supplement: S2 Table — (DOCX) [file pone.0281825.s003.docx]

**S3 Table. Coding framework**

| **Coach injury prevention strategy preferences framework** | | | |
| --- | --- | --- | --- |
| **Core Categories** | **Themes** | **Sub-themes** | |
| **IPP** | Characteristics | Specific exercises included | Flexibility & mobility exercises |
|  |  |  | Fitness and conditioning work |
|  |  |  | Strengthening exercises |
|  |  |  | Plyometrics & agility exercises |
|  |  |  | Fundamental movement patterns |
|  |  | Includes goal setting & progression | |
|  |  | Fun & variety | |
|  |  | Accessible, adaptable, user-friendly | |
|  |  | Includes a warm-up protocol | |
|  |  | Includes a preseason or offseason intervention | |
|  |  | Includes a cooldown protocol | |
|  |  | Sport-specific, games-based, includes the ball | |
|  |  | Targets worst & most common injuries | |
|  |  | Includes several options | |
|  | Type | Individualised IPPs | Specific to an individual’s needs |
|  |  |  | Specific to age group/level |
|  |  | Standardised IPPs | |
|  | Format | Team based with individual elements | |
|  |  | IP completed individually | |
|  | Equipment Use | Anti-/minimal equipment use | |
|  |  | Pro-equipment use | |
|  | Training vs game day use | IPP differs between training and game day | |
|  |  | IPP consistent between training and game day | |
| **Role of stakeholders** | Coaches | Implementing an IPP | |
|  |  | Promoting IP | |
|  |  | Communication | |
|  | Players | Taking some responsibility for their own IP | |
|  |  | Promoting IP | |
|  |  | Providing feedback on IP | |
|  | LGFA | Supporting IP | |
|  |  | Releasing IP strategy | |
|  |  | Pushing IP education | |
|  | Clubs | Supporting IP | |
|  | H&F Professionals | Access to H&F professionals | |
| **Logistics** | IP integrated into sessions | IP at every session | |
|  | IP time required per session | 10-15 minutes of IP | |
|  |  | 16-20 minutes of IP | |
|  |  | 21+ minutes of IP | |
|  | IP begins at a young age | | |
|  | IP completed consistently | | |
| **Guidance and support** | Load management & recovery | Load management & recovery advice | |
|  |  | Load management & recovery policy | |
|  | Injury & IP | Injury & IP advice | |
|  | General Health | Nutrition & hydration advice | |

| **Player injury prevention strategy preferences framework** | | | |
| --- | --- | --- | --- |
| **Core Categories** | **Themes** | **Sub-themes** | |
| **IPP** | Characteristics | Specific exercises included | Strengthening exercises |
|  |  |  | Flexibility & mobility exercises |
|  |  |  | Fundamental movement patterns |
|  |  |  | Fitness and conditioning work |
|  |  |  | Plyometrics & agility exercises |
|  |  | Accessible, adaptable, user-friendly | |
|  |  | Fun & variety | |
|  |  | Includes a warm-up protocol | |
|  |  | Sport-specific, game-based, includes the ball | |
|  |  | Targets worst & most common injuries in LGF | |
|  |  | Includes a preseason or offseason intervention | |
|  |  | Includes goal setting & progression | |
|  |  | Includes several options | |
|  |  | Includes a cooldown protocol | |
|  |  | Evidence-based | |
|  | Type | Individualised IPPs | Specific to an individual’s needs |
|  |  |  | Specific to age group/level |
|  |  | Standardised IPPs | |
|  | Format | Team based IP with individual elements | |
|  |  | IP completed as a team | |
|  | Equipment use | Anti-/minimal equipment use | |
|  |  | Pro-equipment use | |
|  | Training vs game day use | IPP consistent between training and game day | |
|  |  | IPP differs between training and game day | |
| **Role of stakeholders** | Coaches | Implementing IP | |
|  |  | Promoting IP | |
|  | LGFA | Releasing IP strategy | |
|  |  | Pushing IP education | |
|  |  | Supporting IP | |
|  |  | Promoting IP | |
|  | Players | Taking some responsibility for their own IP | |
|  | H&F Professionals | Access to H&F professionals | |
|  |  | Implementing IP | |
|  | Clubs | Supporting IP | |
| **Logistics** | IP integrated into sessions | IP at every session | |
|  | IP time per session | 10-15 minutes of IP | |
|  |  | 16-20 minutes of IP | |
|  |  | 21+ minutes of IP | |
|  | IP begins at a young age | | |
|  | IP completed consistently | | |
| **Guidance and support** | Load management & recovery | Load management & recovery advice | |
|  |  | Load management & recovery policy | |
|  | General Health | Nutrition & hydration advice | |
|  |  | Sleep advice | |

| **Coach injury prevention education preferences framework** | | |
| --- | --- | --- |
| **Core Categories** | **Themes** | **Sub-Themes** |
| **Focus of education** | IP and IP techniques | Why IP is required |
|  |  | IPPs and warm-ups |
|  |  | The benefits of IP |
|  |  | Best practice IP advice |
|  |  | Load management & recovery strategies |
|  |  | Functional movement patterns |
|  |  | The principles of S&C |
|  | The female athlete | Differences between male and female athletes |
|  |  | Menstrual cycle |
|  |  | Anatomy, biomechanics & physiology |
|  |  | Differences across age groups & development |
|  | Injury in LGF | Most common and worst injuries |
|  |  | Risk factors for injury |
|  |  | Impacts of injury |
|  |  | Mechanisms of injury |
|  | Health & lifestyle advice | Nutrition |
|  |  | Hydration |
|  |  | Mental health |
|  |  | Sleep |
| **Format** | Method of delivery | Online education |
|  |  | In-person education |
|  |  | Dual delivery of education |
|  | Accessible to all | |
| **Who needs education** | Coaches | |
|  | Players | |
|  | Clubs | |
|  | LGFA officers | |
|  | Parents | |
| **Time** | Length of education | Bite-size education |
|  |  | 30+min education programme |
|  | Frequency of education | Once/twice a year |
|  |  | Continuous/gradual release |
| **Educator** | H&F professionals | |
|  | Current/past players & coaches | |
|  | LGFA officers | |
| **Roll-out** | Integrated into existing education programmes | |
|  | Educate members & have them spread IP in their clubs | |
|  | H&F professionals/LGFA officers spread IP | |

| **Player injury prevention education preferences framework** | | |
| --- | --- | --- |
| **Core Categories** | **Themes** | **Sub-Themes** |
| **Focus of education** | IP and IP techniques | Why IP is required |
|  |  | IPPs and warm-ups |
|  |  | The benefits of IP |
|  |  | Load management & recovery strategies |
|  |  | Basic or functional movements |
|  | Injury in LGF | Most common and worst injuries |
|  |  | Impacts of injury |
|  |  | Mechanisms of injury |
|  |  | Risk factors for injury |
|  | Health & lifestyle advice | Nutrition |
|  |  | Sleep |
|  |  | Hydration |
|  | The female athlete | Differences between male and female athletes |
|  |  | Anatomy, biomechanics & physiology |
| **Who needs education** | Players | |
|  | Coaches | |
|  | Clubs | |
| **Format** | Method of Delivery | In-person education |
|  |  | Online education |
|  |  | Dual delivery of education |
|  |  | Text-based education |
|  | Accessible to all | |
|  | Interactive | |
| **Educator** | H&F Professionals | |
|  | Current/past players & coaches | |
|  | LGFA officers | |
| **Roll-out** | Educate members & have them spread IP in their clubs | |
|  | H&F professionals/LGFA officers spread IP | |
|  | Integrated into existing education programmes | |
| **Time** | Length of education | Bite-sized education |
|  |  | 30+min education programmes |
|  | Frequency of education | Continuous/gradual release |
|  |  | Once/twice a year |

Note: Categories, themes and sub-themes ordered by most frequently referenced. LGF= ladies Gaelic football, IP= injury prevention, IPP= injury prevention programme, IPS= injury prevention strategy, LGFA= Ladies Gaelic Football Association, H&F= health and fitness, S&C= strength and conditioning.
