# Supplementary material for: A qualitative examination of injury prevention strategy and education in Ladies Gaelic football: Understanding the preferences of players and coaches
Source: PLoS One. 2023 Feb 15;18(2):e0281825. doi: 10.1371/journal.pone.0281825 (PMC9931138; doi:10.1371/journal.pone.0281825)
Supplement: Attachment — Submitted filename: Response to Reviewers.docx [file pone.0281825.s004.docx]

**Response to reviewers**

Thank you so much to both reviewers and the academic editor for taking the time to read and review the manuscript. The comments were very helpful and we appreciate the effort made to do this. We have included responses to each comment below, included any amended text appropriate to the response and also in the manuscript itself highlighted the change in yellow.

**When submitting your revision, we need you to address these additional requirements:**

**1. Please ensure that your manuscript meets PLOS ONE's style requirements, including those for file naming. The PLOS ONE style templates can be found at** 
[**https://journals.plos.org/plosone/s/file?id=wjVg/PLOSOne_formatting_sample_main_body.pdf**](https://journals.plos.org/plosone/s/file?id=wjVg/PLOSOne_formatting_sample_main_body.pdf) **and** [**https://journals.plos.org/plosone/s/file?id=ba62/PLOSOne_formatting_sample_title_authors_affiliations.pdf**](https://journals.plos.org/plosone/s/file?id=ba62/PLOSOne_formatting_sample_title_authors_affiliations.pdf)

The authors have reviewed PLOS ONE’s style requirements and made changes to the formatting of the tables in the manuscript. These changes are highlighted in the manuscript.

**2. Please include your full ethics statement in the ‘Methods’ section of your manuscript file. In your statement, please include the full name of the IRB or ethics committee who approved or waived your study, as well as whether or not you obtained informed written or verbal consent. If consent was waived for your study, please include this information in your statement as well.**

The methods section has been updated to include the following lines:

“This project received ethical approval (#2022/031) from the Dublin City University Research Ethics Committee.”

“informed written consent was obtained prior to commencing data collection”

**3. Please provide additional details regarding participant consent. In the ethics statement in the Methods and online submission information, please ensure that you have specified what type you obtained (for instance, written or verbal, and if verbal, how it was documented and witnessed). If your study included minors, state whether you obtained consent from parents or guardians. If the need for consent was waived by the ethics committee, please include this information.**

The following line has now been updated in the methods section:

“informed written consent was obtained prior to commencing data collection”

and the ethics statement:

“Informed written consent was obtained from all participants prior to commencing data collection.”

**4. In your Data Availability statement, you have not specified where the minimal data set underlying the results described in your manuscript can be found. PLOS defines a study's minimal data set as the underlying data used to reach the conclusions drawn in the manuscript and any additional data required to replicate the reported study findings in their entirety. All PLOS journals require that the minimal data set be made fully available. For more information about our data policy, please see** [**http://journals.plos.org/plosone/s/data-availability**](http://journals.plos.org/plosone/s/data-availability)**.**

**Upon re-submitting your revised manuscript, please upload your study’s minimal underlying data set as either Supporting Information files or to a stable, public repository and include the relevant URLs, DOIs, or accession numbers within your revised cover letter. For a list of acceptable repositories, please see** [**http://journals.plos.org/plosone/s/data-availability#loc-recommended-repositories**](http://journals.plos.org/plosone/s/data-availability#loc-recommended-repositories)**. Any potentially identifying patient information must be fully anonymized.**

**Important: If there are ethical or legal restrictions to sharing your data publicly, please explain these restrictions in detail. Please see our guidelines for more information on what we consider unacceptable restrictions to publicly sharing data:** [**http://journals.plos.org/plosone/s/data-availability#loc-unacceptable-data-access-restrictions**](http://journals.plos.org/plosone/s/data-availability#loc-unacceptable-data-access-restrictions)**. Note that it is not acceptable for the authors to be the sole named individuals responsible for ensuring data access.**

**We will update your Data Availability statement to reflect the information you provide in your cover letter.**

The authors have now provided the data in the supporting information as requested. We have reviewed and updated our data availability statement to reflect this change.

**5. Please include captions for your Supporting Information files at the end of your manuscript, and update any in-text citations to match accordingly. Please see our Supporting Information guidelines for more information:** [**http://journals.plos.org/plosone/s/supporting-information**](http://journals.plos.org/plosone/s/supporting-information)**.**

The authors have added the necessary captions at the end of the manuscript and updated in-text citations where necessary:

“**Supporting information**

S1 File. Interview guide questions for players and coaches.

S2 Table. Standards for Reporting Qualitative Research Checklist.

S3 Table. Coding framework.”

**6. Please review your reference list to ensure that it is complete and correct. If you have cited papers that have been retracted, please include the rationale for doing so in the manuscript text, or remove these references and replace them with relevant current references. Any changes to the reference list should be mentioned in the rebuttal letter that accompanies your revised manuscript. If you need to cite a retracted article, indicate the article’s retracted status in the References list and also include a citation and full reference for the retraction notice.**

The authors have reviewed the reference list and corrected a typo in reference 65 so that it now reads as follows:

“White PE, Otago L, Saunders N, Romiti M, Donaldson A, Ullah S, et al. Ensuring implementation success: how should coach injury prevention education be improved if we want coaches to deliver safety programmes during training sessions? Br J Sports Med. 2014 Mar;48(5):402–3.”

**7. Review Comments to the Author**

**Reviewer #1: I think that the research was written as a structure of the original and journal guidelines. I think that the research it can be said that the article is well written and may contribute to the sports science area.**

**Reviewer #2: The manuscript showed to be well organized in all parts.
The idea behind it is original and well established in other team sports and validated from other studies. Furthermore, I found the manuscript well written.**

The authors greatly appreciate the comments of the reviewers and the time taken to review this manuscript.
